# Effect of elevated depressive symptoms during adolescence on health-related quality of life in young adulthood—a six-year cohort study with repeated exposure measurements

**DOI:** 10.3389/fped.2024.1252964

**Published:** 2024-07-11

**Authors:** Jascha Wiehn, Tobias Kurth, Ulrike Ravens-Sieberer, Christof Prugger, Marco Piccininni, Franziska Reiss

**Affiliations:** ^1^ Institute of Public Health, Charité – Universitätsmedizin Berlin, Berlin, Germany; ^2^ Department of Child and Adolescent Psychiatry, Psychotherapy, and Psychosomatics, University Medical Center Hamburg-Eppendorf, Hamburg, Germany; ^3^ Center for Stroke Research Berlin, Charité – Universitätsmedizin Berlin, Berlin, Germany

**Keywords:** BELLA study, longitudinal, childhood, depression, patient-reported outcomes, physical functioning, observational study, causality

## Abstract

**Objectives:**

Depression is a major contributor of young people's burden of disease. In this study we aim to estimate the effect of elevated depressive symptoms on physical health-related quality of life.

**Design:**

We used self-reported information from the prospective BELLA cohort study, which included adolescents selected from the general population in Germany. The baseline assessment (2003–2006) and the 1-, 2-, and 6-year follow-up waves provide the data basis.

**Participants:**

The baseline study population consisted of 1,460 adolescents between the ages of 12 and 17 who, according to their caregivers, did not suffer from depression.

**Variables:**

The primary outcome, as measured by the physical component score (PCS) of the SF-36 at a 6-year follow-up (range: 0–100), is physical health-related quality of life. The exposure of interest is depressive symptoms, as measured by the Center for Epidemiological Studies Depression Scale for Children (CES-DC) at baseline, 1-year follow-up and 2-year follow-ups (range: 0–60). We dichotomized the exposure into subthreshold (≤15) and elevated depressive symptoms (>15). For the main analyses we considered a cumulative index for elevated depressive symptoms across the three time points (range: 0–3). Considered confounders are sex, age, socioeconomic status, migrant background, social support, anxiety symptoms, physical activity, chronic diseases, and sleeping problems.

**Statistical methods:**

We used multiple imputation to account for missing values. Within each imputed dataset, we applied inverse probability weighting (IPW) to estimate the effect of the cumulative index for elevated depressive symptoms at baseline, 1- and 2-year follow-up on physical health-related quality of life at 6-year follow-up. We derived 95% confidence intervals by bootstrapping.

**Results:**

After adjusting with IPW, the effect of the cumulative index per one unit increase of elevated depressive symptoms on the physical component score was −1.71 (95% CI: −3.51 to −0.04). The adjusted effect estimates of single exposure of elevated depressive symptoms on physical health-related quality of life were −0.83 (95% CI: −3.69 to 1.87) at baseline, −2.96 (95% CI: −4.94 to −0.52) at 1-year follow-up and −1.32 (95% CI: −3.85 to 1.15) at 2-year follow-up.

**Conclusion:**

Findings suggest that elevated depressive symptoms during adolescence decrease physical health-related quality of life in young adulthood.

## Introduction

Adolescence is a critical stage of biological, social, behavioral, and psychological changes (1). Vulnerabilities and resiliencies are developed throughout the transition from infancy to maturity (2). This life stage is the foundation for mental health across the life cycle (3), and accomplishments and setbacks during this period are known to shape young people's personalities and wellbeing (4, 5).

At this vulnerable age, depressive symptoms can increase rapidly (6). Thus, if elevated depressive symptoms are to be detected, screening should ideally be repeated at regular intervals (7). Elevated depressive symptoms are characterized by the loss of energy, interest, or pleasure (anhedonia) and the continuous feeling of being sad (low mood) (8). It is estimated that between 30% and 38% of adolescents worldwide suffer from elevated depressive symptoms (9). Experiencing elevated levels of depressive symptoms in adolescence is linked to various adverse health outcomes in young adulthood (10, 11). Among the adverse health outcomes is lower health-related quality of life (12), which can be defined as “how well a person functions in their life and his or her perceived wellbeing in physical, mental, and social domains of health” (13).

Numerous studies have shown that elevated depressive symptoms are associated with lower health-related quality of life. However, many of these studies have recruited adult (14–29), clinical (16, 18–23, 25–28, 30–38), or community samples (14, 15, 24, 29, 39, 40), with limited generalizability. Rarely studies have included children and adolescents from the general population (41). Moreover, it may be challenging to establish causal relationships using cross-sectional data (14–22, 24–27, 29–41), and the few longitudinal studies that exist, recruited at risk youths (42), adult clinical (28) or pregnant samples (23). One cohort study sampling adolescents, found that the elevated depressive symptoms may precede the health-related quality of life domains “ability to cope” and “social relationships”, but not overall health-related quality of life (43). However, the authors did not investigate whether elevated depressive symptoms affect physical health-related quality of life.

Studying the impact of depressive symptoms during adolescence on the physical health-related quality of life in young adulthood holds significant potential in unraveling the factors that influence the well-being of young individuals. This research can provide public health professionals with valuable insights on the physical benefits of good mental health and help them to make informed decisions pertaining to the allocation of resources for prevention strategies that target both mental and physical health. Our study aimed at quantifying the effect of elevated depressive symptoms during adolescence on the physical component of health-related quality of life in young adulthood.

## Methods

### Study design

The present study is based on longitudinal data from the BELLA cohort study (44). The BELLA study is the mental health module of the German National Health Interview and Examination Survey for Children and Adolescents (KiGGS), which was prepared and conducted by the Robert Koch Institute (Berlin, Germany). The KiGGS study aimed to enroll children and adolescents aged 0–17 years living in Germany, based on their official residence listed in local registration offices (45).

**Figure 1:**
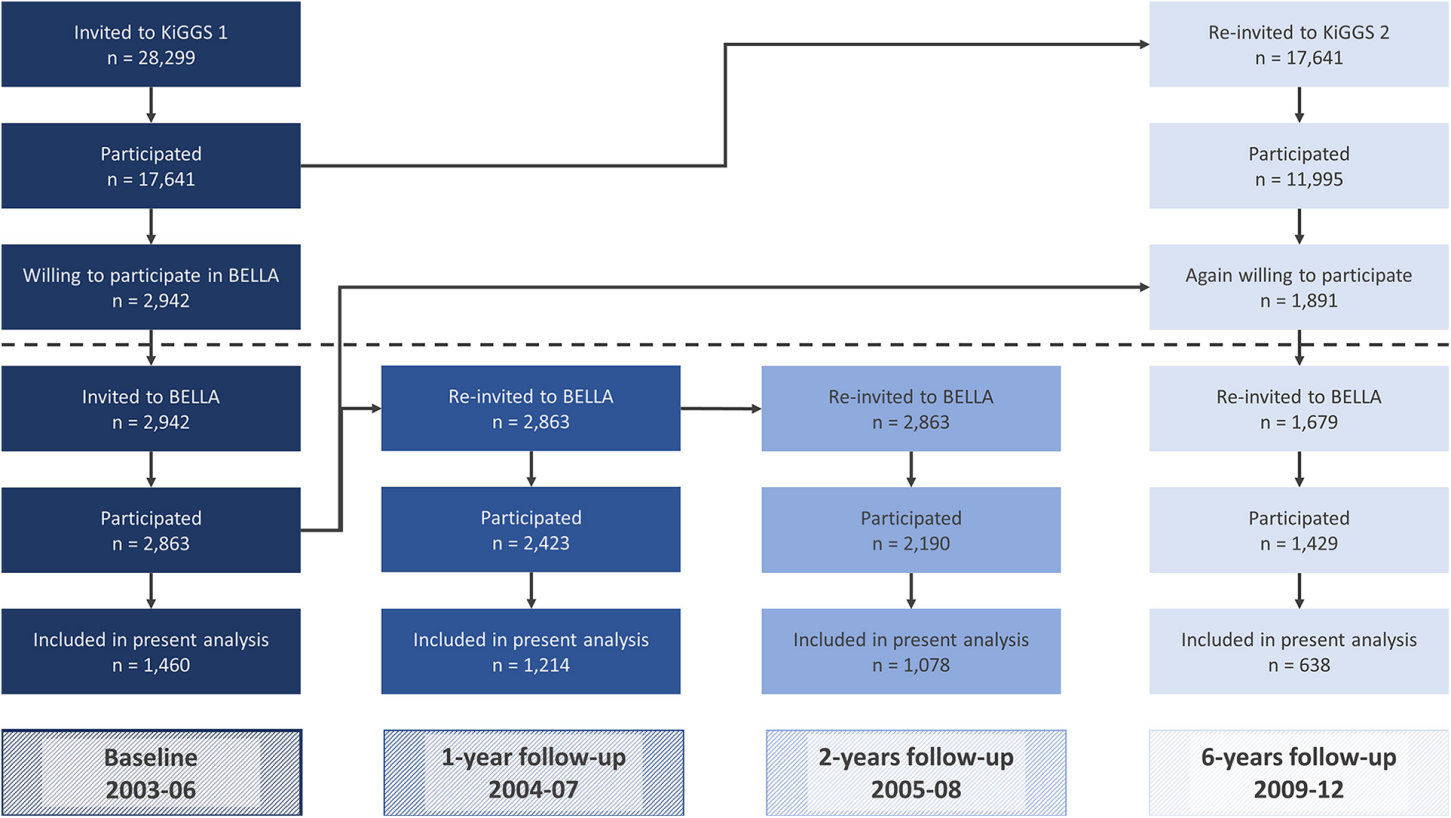
Flow of participants of the KIGGS and the BELLA study as well as participants in the present analysis.

As part of the KiGGS study, the BELLA study is a population-based, nationwide cohort study that collects data on mental health and well-being of children and adolescents in Germany. The baseline assessment took place between 2003 and 2006 (see Figure 1). Three follow-up waves have been conducted one year (2004–2007), two years (2005–2008), six years (2009–2012) and eleven years (2014–2017) after the baseline data collection. This analysis is based on the baseline assessment and the first three follow-up waves. For the BELLA 6-year follow-up, the KiGGS study team had to request caregivers to give an additional informed written consent to be invited, due to new German data protection regulations. 1,891 families gave their consent. The BELLA study team then re-invited 1,679 subjects and 1,429 of those participated at the 6-year follow-up. For further details on the design and procedure of the BELLA or the KiGGS study, please refer to previously published methodology reports (44–46).

We specified our population of interest based on three inclusion and exclusion criteria: First, adolescents had to participate at baseline. Second, participants had to be aged 12–17 years at baseline. Third, adolescents should not have a parent-reported current or former depression at baseline.

Prior to data collection, written informed consent was obtained from adolescents (aged 14 years or older) and parents to participate in the study. Participation in the survey was voluntary, and respondents could cancel their participation at any time without having to give reasons. The ethics committee of the Charité – Universitätsmedizin Berlin and the Federal Commissioner for Data Protection in Germany approved the study (44).

### Variables

#### Health-related quality of life

The primary outcome of the present study is physical health-related quality of life at 6-year follow-up (18–23 year olds), as measured by the German translation of the version 2.0 of the 36-Item Short Form Survey (SF-36) (47–50). The SF-36 is the most widely used generic instrument to assess health-related quality of life in the general population (51, 52). It is a self-reported multidimensional instrument with 36 items to depict vitality, bodily pain, general health perceptions, mental health, physical functioning, physical role functioning, emotional role functioning, and social role functioning. These eight health domains yield in two summary scores: the physical component score (PCS) and the mental component score (MCS) (53). Out of 36 items, 21 items are summed to calculate the PCS and 14 items are summed to calculate the MCS. The item that measures how general health status changed over the past year is not applied. Both summary scores can range from 0 to 100, with a higher score indicating better quality of life within the last four weeks. In this investigation, we rely on the PCS as the outcome of interest. Both the original English and the German version of the SF-36 showed good psychometric proprieties in terms of validity and reliability (49, 54–56).

#### Depressive symptoms

The exposure of interest was depressive symptoms, as measured by self-reported depressive symptoms assessed at baseline, 1- and 2-year follow-ups using the German version of the Center for Epidemiological Studies Depression Scale for Children (CES-DC) (57–59). The CES-DC is a widely-used self-reported screening tool that measures six domains of depressiveness through 20 items (60). The CES-DC is adapted from the CES-D, which is a depression screening tool for adults (61). The items of the CES-DC refer to depressive symptoms experienced in the past week, and responses are recorded on a four-point Likert scale. Four of the items are phrased positively and are reverse-coded for scoring. The total score ranges from 0 to 60, with higher scores indicating greater depressiveness. The German CES-DC has been shown to be a reliable and valid instrument, and self-reported data is considered more valid than parental information (59). To analyze the data, the CES-DC scores at each exposure time point were dichotomized using the Weissmann cut-off of >15 (58), which is optimal for screening children and adolescents for current major depressive disorder or dysthymia (62). For the modelling, we summarized the exposure at the three measurement times into a cumulative exposure index cum(ā) counting the number of times the participant had a CES-DC score higher than the threshold. This results in a range from 0 (never elevated depressive symptoms) to 3 (elevated depressive symptoms at all three measurement points).

#### Covariates

**Figure 2:**
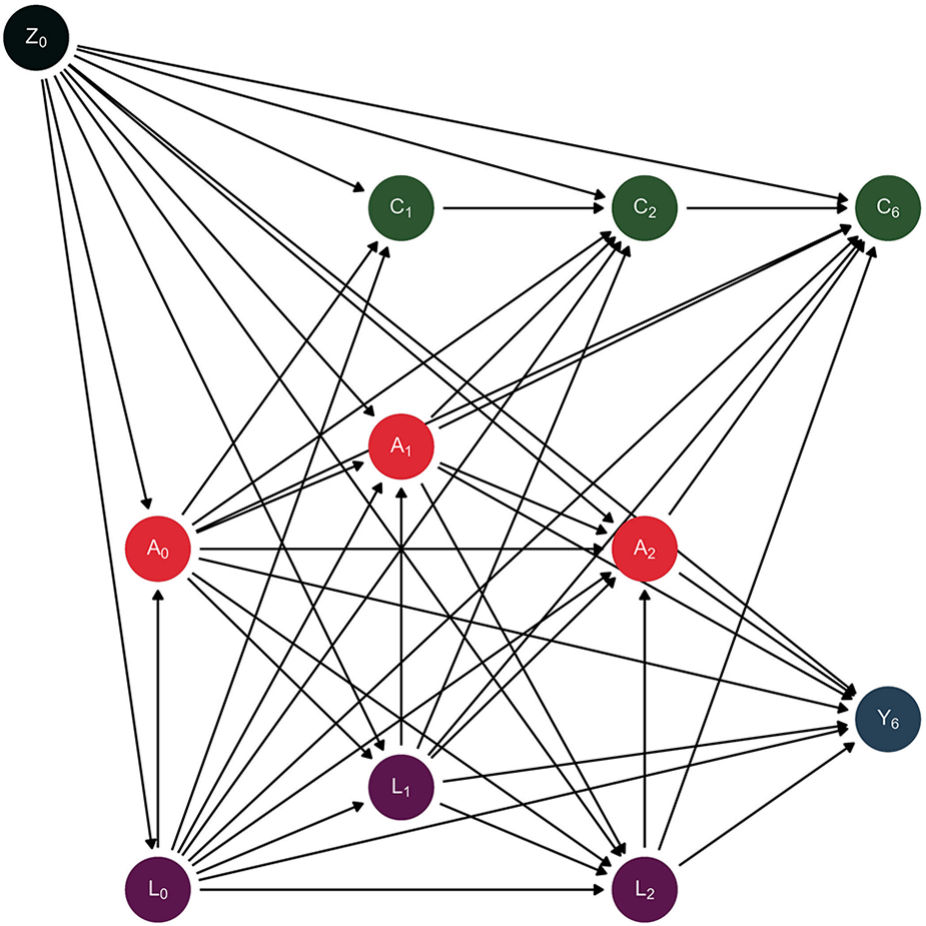
Directed acyclic graph depicting the assumed data generation process. Causal direct effects are represented by arrows.

To identify confounding variables, we drew a directed acyclic graph (DAG) to represent our assumptions about the data generation process (63). The DAG is schematically represented in Figure 2. We identified both time-fixed confounders (Z) and time-varying confounders (L). Further information about the use of DAGs can be found elsewhere (63–68).

We considered as time-fixed confounders the following variables: biological sex (male, female), socioeconomic status (SES-Index, range: 3–21, measured by parental education, household income and parental employment) (69), migrant background (yes, no) (70), and age (years). These parent-reported covariates were measured at baseline. We considered as time-varying confounders the following variables: anxiety symptoms (range: 0–10; The Screen for Child Anxiety Related Emotional Disorders – SCARED-5) (71, 72), social support (range: 1–5; Social Support Survey) (73, 74), physical activity (hours per week), chronic diseases or disability (yes, no), and sleeping problems (yes, no). These time-varying confounding variables were all self-reported and measured at baseline, 1- and 2-year follow-up waves. Finally, censoring (C) is also depicted as non-participation (yes, no) at 1-, 2- and 6-year follow-up.

The graph visualizes the assumed causal relationship between elevated depressive symptoms (A) and physical health-related quality of life (Y) as well as time-fixed confounders (biological sex, socioeconomic status, migrant background, age; Z), time-varying confounders (anxiety symptoms, social support, physical activity, chronic diseases or disability, sleeping problems; L) and censoring (non-participation; C). The subscript numbers correspond to the respective time of measurement at baseline (Z_0_, A_0_, L_0_), 1-year follow-up (C_1_, A_1_, L_1_), 2-year follow-up (C_2_, A_2_, L_2_) or 6-year follow-up (C_6_, Y_6_).

### Statistical methods

Our statistical analysis consisted of three main steps. First, we used multiple imputation to handle missing information due to non-response. Second, within each of the imputed datasets we used inverse probability weighting (IPW) to account for exposure-confounder-feedback and differential loss to follow-up. Third, we estimated the unadjusted and adjusted effect of different patterns of depressive symptoms over a period of two years on the physical component of health-related quality of life at six years follow-up.

#### Multiple imputation

Overall, 22% of the raw data was missing (see Supplementary S1 for the proportion of missing values in each variable). Complete data for all time points was available for 250 individuals. Information was either missing because (i) participants did not attend the follow-up (non-participation) or (ii) they did not provide information when they attended (non-response). Under a missing at random (MAR) assumption (75), we used multiple imputation by chained equations (76) with five imputed datasets to impute the missing values for individuals who were not lost to follow up but did not give information (non-response). In order to create a closed cohort, we considered a participant lost to follow-up if he or she did not participate in a visit, regardless of participation to following visits, even if this caused a loss of information. We imputed missing values due to non-response for those subjects who were not lost to follow-up using the baseline variables of sex, age, socioeconomic status, migrant background, social support, anxiety symptoms, physical activity, chronic diseases, sleeping problems, and depressive symptoms. After imputing missing values, the overall percentage of missing data in the whole dataset decreased to 15.3%. We could therefore include 546 complete cases in the main analysis.

#### Inverse probability weighting

Then, for each of the five imputed data sets, IPW was utilized. In order to quantify the probability of elevated depressive symptoms at baseline, 1- and 2-year follow-up, we fitted three logistic regression models. In the first model, we utilized baseline covariates as predictors for the baseline exposure. For the 1- and 2-year follow-up models, we fit the regressions among participants not lost to follow-up and included all covariates of the respective and the previous waves as well as elevated depressive symptoms (binary) from earlier waves. We used the coefficients of these three regression models to calculate the probability of reporting elevated vs. subthreshold depressive symptoms at each time point and for each individual. To calculate the probability of being lost to follow-up at 1-, 2-, and 6-year follow-ups, we fitted three further logistic regression models using all available data on covariates and exposure from the previous waves.

We then calculated the weights for the exposure for each individual and each time point as the inverse of the probability of being in their levels of depressive symptoms (elevated vs. subthreshold) at the given time point (W_A0_, W_A1_, W_A2_). Subsequently, we calculated the weights for the censorship as zero for individuals who are lost to follow-up and the inverse of the probability of not being lost to follow-up at the given time point for individuals not lost to follow-up (W_C1_, W_C2_, W_C6_). We multiply these six weights to gain a single weight (W). For the subsequent effect estimation, this single weight variable was applied to each subject in the weighted regression models within the five imputed datasets.

#### Effect estimation

First, we estimated the unadjusted association between the cumulative exposure index and the physical component score at 6-year follow-up (Y_6_) (model 1). We assessed the effect of this cumulative exposure index on the outcome using a linear regression model. We therefore assumed that the expected physical component score is a linear function of the cumulative index for elevated depressive symptoms, regardless of the specific symptom pattern (63).

In the first weighted linear regression model, we estimated the effect of cumulative exposure index on the physical component score at 6-year follow-up (Y_6_) (model 2). In the second weighted regression model, we estimated the effect of the different depression symptoms patterns on the physical component score at 6-years considering the three exposure statuses as single binary variables rather than relying on the cumulative exposure index (model 3). Point estimates were obtained by averaging the estimated coefficients across the five imputed datasets. For all models, we ran 500 iteration bootstraps to obtain 95% confidence intervals of the coefficients (CI) (77).

We used R version 4.2.1 and RStudio version 2022.12.0 for data analysis.

## Results

In total, 2,863 children and adolescents aged 7–17 years, participated at the baseline of the BELLA study (Figure 1). With reference to our inclusion criteria, we excluded 1,394 participants aged 11 years and younger at baseline, four adolescents whose parents indicated that their kids were currently depressed at baseline and another five youths whose parents reported that their kids were formerly depressed at baseline. This resulted in a final baseline sample of 1,460 adolescents aged 12–17 years at baseline (Table 1).

**Table 1:** Baseline characteristics of study participants.

Sample characteristics	Total (*n* = 1,460)
Biological sex, *n* (%)	
Male	724 (49.6%)
Female	736 (50.4%)
Missing	–
Socioeconomic status	
Mean (SD)	11.7 (4.2)
Missing	11 (0.9%)
Migrant background, *n* (%)	
Yes	151 (10.3%)
No	1,308 (89.6%)
Missing	1 (0.1%)
Age in years	
Mean (SD)	14.9 (1.7)
Missing	–
SCARED-5	
Mean (SD)	15.2 (8.5)
Missing	41 (2.8%)
Social support	
Mean (SD)	4.1 (0.7)
Missing	7 (0.5%)
Physical activity (hours/week)	
Mean (SD)	7.3 (7.6)
Missing	224 (15.3%)
Chronic diseases or disability, *n* (%)	
Yes	173 (11.8%)
No	1,245 (85.3%)
Missing	42 (2.9%)
Sleeping problems, *n* (%)	
Yes	294 (20.1%)
No	1,160 (79.5%)
Missing	6 (0.4%)

n, number of observations; SD, standard deviation; SCARED-5, the Screen for Child Anxiety Related Emotional Disorders.

### Exposure data

Mean depressive symptoms at baseline were 10.1 (SD: 7.0). After dichotomization (CES-DC > 15), 251 out of 1,460 participants (17.2%) were classified as showing elevated levels of depressive symptom at the beginning of the study. The proportion of adolescents with elevated depressive symptoms was 11.5% at the 1-year follow-up and 9.7% at the 2-year follow-up. During the baseline, 1-year and 2-year follow-up surveys, 602 of 1,460 adolescents (41.2%) reported depressive symptoms below the threshold at all three time points. Another 173 youths reported elevated levels of depressive symptoms at one of the three assessment points (11.8%), 63 at two assessment points (4.3%) and 33 at all three assessment points (2.3%).

### Outcome data

**Figure 3:**
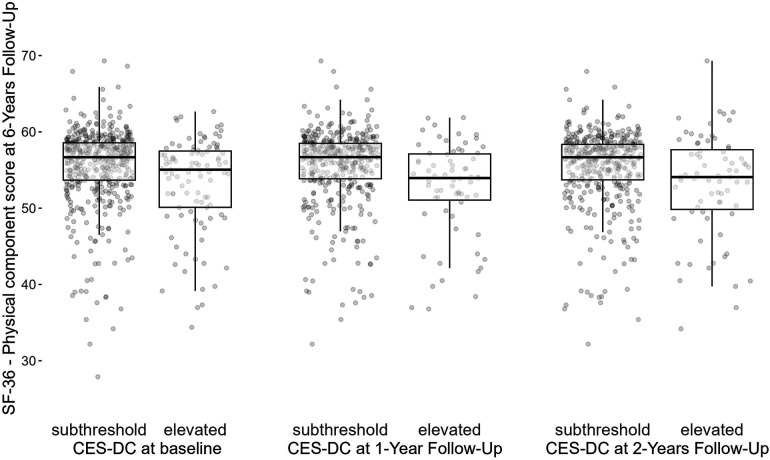
Physical component score of the SF-36 at 6-year follow-up by elevated levels of depressive symptoms (>15) at baseline, 1- and 2-year follow-up waves. CES-DC, the Center for Epidemiological Studies Depression Scale for Children; SF-36, 36-Item Short Form Survey.

At 6-year follow-up, the physical component score of the SF-36 had a mean of 55.1 (SD: 5.5, missing: 59%). Figure 3 shows that participants reporting lower depressive symptoms at baseline on average had better physical health-related quality of life scores at 6-year follow-up compared to those reporting elevated depressive symptoms at baseline (mean: 55.5, SD: 5.1 vs. mean: 53.2, SD: 6.2). Higher levels of the physical component score on average were also observed among subjects with subthreshold depressive symptoms at 1-year follow-up (mean: 55.5, SD: 5.0 vs. mean: 52.6, SD: 6.2) as well as at 2-year follow-up (mean: 55.4, SD: 5.0 vs. mean: 53.0, SD: 6.8).

### Main results

As depicted in Table 2, the unadjusted association between the cumulative index for elevated depressive symptoms and physical component score of SF-36 at 6-year follow-up was −1.49 [95% confidence interval (CI): −2.18 to −0.83] (model 1). After adjusting for confounding and loss to follow-up via IPW, the effect of the cumulative exposure index cum(Ā) on the physical component score was −1.71 (−3.51 to −0.04) (model 2). In model 3, all coefficients were negative and the null value was not included in the confidence interval for the effect estimate of the exposure at 1-year follow-up.

**Table 2:** Unadjusted and adjusted effect estimates of depressive symptoms on physical component score of SF-36.

Model	Mean overall weight of each imputed dataset (min, max)						Estimates of *ß*			
	1	2	3	4	5	Intercept (95% CI)	cum(Ā) (95% CI)	CES-DC_0_ (95% CI)	CES-DC_1_ (95% CI)	CES-DC_2_ (95% CI)
Model 1 (unadjusted)	–	–	–	–	–	55.77 (55.28 to 56.24)	−1.49 (−2.18 to −0.83)	–	–	–
Model 2	9.96 (0, 671.10)	10.06 (0, 808.69)	10.31 (0, 935.79)	9.94 (0, 733.16)	10.15 (0, 984.52)	56.34 (54.01 to 58.23)	−1.71 (−3.51 to −0.04)	–	–	–
Model 3	9.96 (0, 671.10)	10.06 (0, 808.69)	10.31 (0, 935.79)	9.94 (0, 733.16)	10.15 (0, 984.52)	56.26 (54.00 to 58.18)	–	−0.83 (−3.69 to 1.87)	−2.69 (−4.94 to −0.52)	−1.32 (−3.85 to 1.15)

min, minimum; max, maximum; CI, confidence interval; CES-DC, The Center for Epidemiological Studies Depression Scale for Children; SF-36, 36-Item Short Form Survey.

Model 1: Unadjusted associations of cumulative index for elevated depressive symptoms [cum(Ā)] on physical component score of SF-36; Model 2: Adjusted effect estimate of cumulative index for elevated depressive symptoms on physical component score of SF-36 based on inverse probability weighting (IPW); Model 3: Adjusted effect estimates of elevated depressive symptoms at single waves on physical component score of SF-36 based on IPW. The mean, minimum, and maximum of each imputed data set's overall weights are presented for models 2 and 3, which employ IPW.

## Discussion

### Key results and interpretations

Our research aimed to estimate the effect of different patterns of elevated depressive symptoms during adolescence on the physical component of health-related quality of life in young adulthood. Our adjusted analyses showed that the average physical quality of life of young adults decreased by 1.7 units with each measurement time point they reported elevated depressive symptoms. This suggests that the physical health-related quality of life tends to decline as adolescents show depressive symptoms above the threshold for a longer period. Our analysis showed that young individuals who experienced elevated depressive symptoms at all time points had an average physical component score of 51.2. This score is 5.1 points lower compared to the expected score of young people who did not experience depressive symptoms at any time point (56.3).

We found a statistically significant association between elevated depressive symptoms at 1-year follow-up and physical health-related quality of life when we relaxed the assumption of linearity of the effect of the cumulative exposure index. However, there was no statistically significant association between baseline and 2-year follow-up levels of elevated depressive symptoms and the outcome. This may be explained by the fact that relaxing the linearity assumption requires the estimation of more parameters, which ultimately reduces statistical power.

### Comparison with other observational studies

#### Cross-sectional studies

In line with our results, prior cross-sectional studies found that elevated depressive symptoms were statistically associated with physical health related quality of life in adolescents (32, 35, 39–41) and adults (15–17, 21). On the contrary, in one cross-sectional study in adults, no evidence of association was found (19). According to the authors, this could be due to the fact that their clinical sample included participants with Friedreich's ataxia suffering from severely impaired physical functioning. Depending on the timing of depressive symptoms and physical health-related quality of life, cross-sectional studies may not provide a causal estimate as both are measured at a single time point and these variables affect each other in a complex way. Nevertheless, cross-sectional data can provide meaningful information in terms of generating (causal) hypothesis (78, 79).

#### Longitudinal studies

Several longitudinal studies have been conducted. These studies provide additional evidence supporting the relationship between depressive symptoms and health-related quality of life, although each study has its own unique focus, methodology, and limitations.

In a clinical multicenter cohort study in the United States, the relationship between depressive symptoms and health-related quality of life was examined in 147 adults with epilepsy (28). The study found that depressive symptoms accounted for a significant portion of the variation in health-related quality of life in adult epilepsy patients. However, the limitations of the study, including its descriptive nature, clinical sample, disease-specific measurement instrument, and chosen statistical analysis, restrict the comparability of the findings to other studies.

An Irish longitudinal study aimed to investigate the relationship between health-related quality of life, depressive symptoms, body mass index (BMI), and weight change desires in children and adolescents (42). The study suggested that elevated depressive symptoms mediated the link between weight change desires and health-related quality of life. Despite differences in sample, measurement tools, and analysis methods, the study supported the directionality of the association between depressive symptoms and health-related quality of life observed in the current study.

A Taiwanese cohort study examined the mediating role of depressive and anxiety symptoms in the relationship between attention-deficit/hyperactivity disorder (ADHD) symptoms and quality of life, but also whether depressive symptoms may account for poorer quality of life in adolescents (80). The study found that elevated depressive symptoms at baseline resulted in a decrease in health-related quality of life at the 1-year follow-up. Although the study differed in objectives, measurements, and analysis, it provided additional support for the finding that elevated depressive symptoms contribute to poorer overall quality of life in adolescents.

An Australian cohort study of 1,393 adolescents aged 11–14 examined the bi-directional relationship between depressive symptoms, health-related quality of life, and eating disorder symptoms over one year (43). The study found that elevated depressive symptoms at baseline predicted certain aspects of health-related quality of life at the 1-year follow-up. However, the study did not address physical health-related quality of life, adjust for confounders, consider repeated exposure to depressive symptoms, or have the same time interval between exposure and outcome as the current study.

Another US cohort study incorporated variability of depressive symptoms over time to estimate the effect of change in depressive symptoms on health-related quality of life during and after pregnancy (23). The study identified different groups based on patterns of depressive symptoms over time and found a decline in physical health-related quality of life with elevated depressive symptoms. Although the study's focus on pregnant women limits direct comparison to the current study, it provides valuable insights into the impact of depressive symptoms on health-related quality of life.

### Strengths

Through our study, we aim to advance the current knowledge and provide robust evidence regarding the causal nature of the relationship between depressive symptoms and health-related quality of life.

Our study stands out from prior descriptive and predictive studies (81, 82) or those that remain vague and ambiguous in their objectives (83) by explicitly formulating a causal question. Many studies in the field of psychology, for instance, tend to avoid explicit causal inference when working with observational data. For example, researchers investigating psychological topics often avoid explicit causal inference on the basis of observational data (84). While they may not use causal language in their objectives, they often implicitly draw causal implications in their discussions or solely refer to the associational nature of their findings (85). Therefore, their added value in terms of informing interventions and making public health decisions remains unclear (86).

To reduce the risk of tautological inferences, we defined physical health-related quality of life as our primary outcome, because mental health-related quality of life (15, 29, 87) and overall health-related quality of life (40, 88) (which includes a mental component) may conceptually and empirically overlap with the measurement of depressive symptoms (89–93).

We used data from the BELLA cohort study with repeated measures of exposure and confounding variables. This allows us to establish a causal relation and model the dynamic and time dependent nature of the data structure across multiple time points. Furthermore, the data consists of information provided by adolescents drawn from the general population, which strengthens the external validity of our results.

We used state-of-the-art causal methods. That is, we drew a DAG (82) to make our assumptions about the underlying causal structure explicit and open to scrutiny (94). Moreover, we applied IPW to addresses confounding and selection bias, including exposure-confounder-feedback and differential loss to follow-up (63, 95, 96). DAGs can help to identify variables to fulfill the conditional exchangeability assumption, enabling us to use IPW to validly estimate the average causal effect of different exposure patterns despite the presence of treatment confounding feedback (63).

Finally, we used multiple imputation to impute missing values, thus reducing potential risks of non-response bias and increasing statistical power (97, 98).

### Limitations

Advocates of the interventionist causal framework may argue that elevated depressive symptoms is an ill-defined exposure (86, 99, 100). This is because having elevated depressive symptoms is a state rather than a manipulable intervention. It is possible to conceive different interventions that alter the depressive symptoms, with possibly different effects on the outcome. As a result of this violation of the consistency identification assumption (63, 100), the causal effect of having elevated depressive symptoms can be hard to define in counterfactual terms. This challenge is not unique to our study but applies also to previous research on depressive symptoms and health-related quality of life, as well as other studies examining non-manipulable etiological factors (101–104). We take a pragmatic stance and acknowledge that elevated depressive symptoms may represent an ill-defined intervention in terms of the potential outcome framework. Although our results do not provide a precise indication of which interventions contribute to enhancing physical health related quality of life, they are useful to make a general case for early depression prevention.

A relatively small number of participants took part in all follow-up waves and some participants had missing values for some relevant variables. We relied on IPW to address differential loss to follow-up and used multiple imputation by chained equations to impute missing values. Despite the use of state-of-the-art methods to account for non-participation and non-response, the limited sample size reduced our statistical power, increasing the degree of uncertainty of our findings. We emphasize, however, that a small sample size should not be used as an argument to dismiss analysis of observational data for relevant causal questions (105).

For the results of our statistical analysis to be valid, we must have correctly specified the models and correctly identified the confounding variables (106). For example, we assumed that drinking (107, 108) and smoking (109, 110) are not a cause but only a consequence of depressive symptoms. These health risk behaviors were therefore not conceived as confounders but as mediators on the path from depressive symptoms to physical health-related quality of life. Despite employing directed acyclic graphs, it is impossible to verify if the conditional exchangeability assumption is met. We need to rely on our expert knowledge and put our set of covariates up for scientific discussion.

Even if we accurately specified our adjustment set, measurement errors could still result in bias. For example, in the BELLA study, the level of physical activity per week was assessed using a single self-reported item. This measurement may not accurately reflect the true level of physical activity due to recall and social desirability bias. This is a general issue in observational studies interested in physical activity. In recent years, “next generation” epidemiologic studies started utilizing wearable devices to measure physical activity and sedentary behavior, instead of relying on self-reported assessments (111). Similarly, misclassifying the exposure, outcome, or confounder status could lead to information bias in our study. For instance, adolescents with elevated depressive symptoms may be more concerned about their health status, which could cause them to report the physical health consequences of their depressive symptoms more accurately or report worse health consistently with a more negative view. Another source of measurement error in our study is the covariate socioeconomic status, which we measured only at baseline and which we assumed to be constant during the follow-up waves. Of course, changes in parents' education, job promotions, or job loss could result in misclassification of socioeconomic status during the follow-up period. However, given the short duration of our follow-up period and the relatively stable social mobility in Germany, we assume that any variations in socioeconomic status during the follow-up period would be negligible. In support of this, data from the German Socio-Economic Panel suggest that the total variance of individual income trends in Germany between 2008 and 2013 was zero (112).

### Generalizability and implications

Our study sample is a subsample of the KiGGS study population, which aimed at enrolling a representative sample of children and adolescents aged 0–17 years living in Germany (45). For this reason, we consider our findings to be generalizable to the population of adolescents living in Germany aged 12–17 years. Taking into account cultural and social factors, we believe that our findings may also be transportable to similar settings (e.g., other Central European countries).

Numerous systematic reviews and meta-analyses have shown that early prevention interventions, especially selective and indicated programs, can reduce depressive symptoms in children and adolescents (113–124). For example, Wijnhoven et al. showed that in schoolgirls aged 11–15 years with elevated depressive symptoms, cognitive behavioral therapy sessions were effective in reducing depressive symptoms during the intervention and at 6-month follow-up compared to no treatment (125). Our results suggest that physical health-related quality of life increases each time depressive symptoms remain below the threshold. Therefore, if adolescents with elevated depressive symptoms receive interventions such as cognitive behavioral therapy, this could have a positive impact not only on their mental health, but also on their physical health-related quality of life in young adulthood.

Indeed, our results are compatible with the idea that these interventions may have some co-benefits: if interventions can prevent adolescents from experiencing elevated depressive symptoms, this may not only serve the mental health of these adolescents but can also have an impact on their physical health-related quality of life. We refer to the added physical health gains over and above mental health as the physical co-benefits of depression prevention in adolescence. In practical terms, these physical co-benefits could give additional justification to preventive interventions since the overall health gains could outweigh the economic costs of early mental health interventions.

## Conclusion

In this study, we found that elevated depressive symptoms in adolescence reduce physical health-related quality of life in young adulthood. Our results support that health-related quality of life is the product of the dynamic interplay between mental health and physical health-related factors.

## Data Availability

The datasets presented in this article are not readily available because their use is restricted to non-commercial purposes. Requests to access the datasets should be directed to FR, f.reiss@uke.de.
