# Supplementary material for: Effect of elevated depressive symptoms during adolescence on health-related quality of life in young adulthood—a six-year cohort study with repeated exposure measurements
Source: Front Pediatr. 2024 Jul 11;12:1252964. doi: 10.3389/fped.2024.1252964 (PMC11269264; doi:10.3389/fped.2024.1252964)
Supplement: Supplementary file 1 [file Table1.docx]

Supplementary Material

# Absolute and relative number of missing values in each variable by decreasing order.

| **Variable** | **Before MI** | | **After MI** | |
| --- | --- | --- | --- | --- |
|  | **N** | **(%)** | **N** | **(%)** |
| Physical component score (6-year follow-up) | 861 | (59.0) | 828 | (56.7) |
| Physical activity (2-years follow-up) | 778 | (53.3) | 397 | (27.2) |
| Physical activity (1-year follow-up) | 630 | (43.2) | 246 | (16.8) |
| Social support (2-years follow-up) | 627 | (42.9) | 397 | (27.2) |
| Sleeping problems (2-years follow-up) | 617 | (42.3) | 397 | (27.2) |
| SCARED-5 (2-years follow-up) | 468 | (32.1) | 397 | (27.2) |
| Chronic diseases or disability (2-years follow-up) | 468 | (32.1) | 397 | (27.2) |
| CES-DC (2-year follow-up) | 468 | (32.1) | 397 | (27.2) |
| Sleeping problems (1-year follow-up) | 459 | (31.4) | 246 | (16.8) |
| Social support (1-year follow-up) | 451 | (30.9) | 246 | (16.8) |
| Chronic diseases or disability (1-year follow-up) | 414 | (28.4) | 246 | (16.8) |
| SCARED-5 (1-year follow-up) | 412 | (28.2) | 246 | (16.8) |
| CES-DC (1-year follow-up) | 411 | (28.2) | 246 | (16.8) |
| Physical activity (baseline assessment) | 224 | (15.3) | – | – |
| Chronic diseases or disability (baseline assessment) | 42 | (2.9) | – | – |
| CES-DC (baseline assessment) | 42 | (2.9) | – | – |
| SCARED-5 (baseline assessment) | 41 | (2.8) | – | – |
| Socioeconomic status (baseline assessment) | 17 | (1.2) | – | – |
| Social support (baseline assessment) | 7 | (0.5) | – | – |
| Sleeping problems (baseline assessment) | 6 | (0.4) | – | – |
| Migrant background (baseline assessment) | 1 | (0.1) | – | – |
| Biological sex (baseline assessment) | – | – | – | – |
| Age in years (baseline assessment) | – | – | – | – |
